# Prolonged pregnancy and stillbirth among women with overweight or obesity – a population-based study in Sweden including 64,632 women

**DOI:** 10.1186/s12884-022-05340-4

**Published:** 2023-01-12

**Authors:** Anna Akselsson, Jenny Rossen, Elisabeth Storck-Lindholm, Ingela Rådestad

**Affiliations:** 1 Department of Health Promoting Science, Sophiahemmet University, 5605, S-114 86 Stockholm, PB Sweden; 2 Department of Health Promoting Science, Sophiahemmet University, Stockholm, Sweden; 3 Department of Obstetrics and Gynecology, Stockholm South General Hospital, Stockholm, Sweden; 4 Sophiahemmet University, Stockholm, Sweden

**Keywords:** Overweight, Obesity, Pregnancy, Stillbirth, Body Mass Index, Pregnancy outcomes

## Abstract

**Background:**

The proportion of overweight or obese pregnant women is increasing in many countries and babies born to a mother who is overweight or obese are at higher risk for complications. Our primary objective was to describe sociodemographic and obstetric factors across Body Mass Index (BMI) classifications, with secondary objective to investigate stillbirth and other pregnancy outcomes in relation to BMI classifications and gestational week.

**Methods:**

This population-based cohort study with data partly based on a cluster-randomized controlled trial includes 64,632 women with singleton pregnancy, giving birth from 28 weeks’ gestation. The time period was January 2016 to 30 June 2018 (2.5 years). Women were divided into five groups according to BMI: below 18.5 underweight, 18.5–24.9 normal weight, 25.0–29.9 overweight, 30.0–34.9 obesity, 35.0 and above, severe obesity.

**Results:**

Data was obtained for 61,800 women. Women who were overweight/obese/severely obese had lower educational levels, were to a lesser extent employed, were more often multiparas, tobacco users and had maternal diseases to a higher extent than women with normal weight. From 40 weeks’ gestation, overweight women had a double risk of stillbirth compared to women of normal weight (RR 2.06, CI 1.01–4.21); the risk increased to almost four times higher for obese women (RR 3.97, CI 1.6–9.7). Women who were obese or severely obese had a higher risk of almost all pregnancy outcomes, compared to women of normal weight, such as Apgar score < 7 at 5 min (RR1.54, CI 1.24–1.90), stillbirth (RR 2.16, CI 1.31–3.55), transfer to neonatal care (RR 1.38, CI 1.26–1.50), and instrumental delivery (RR 1.26, CI 1.21–1.31).

**Conclusions:**

Women who were obese or severely obese had a higher risk of almost all adverse pregnancy outcomes and from gestational week 40, the risk of stillbirth was doubled. The findings indicate a need for national guidelines and individualized care to prevent and reduce negative pregnancy outcomes in overweight/obese women. Preventive methods including preconception care and public health policies are needed to reduce the number of women being overweight/obese when entering pregnancy.

**Supplementary Information:**

The online version contains supplementary material available at 10.1186/s12884-022-05340-4.

## Introduction

Obesity is one of the most significant contributors to ill health [1] and the number of pregnant women who are overweight or obese is increasing in many countries [2, 3]. Babies born to a mother who is overweight or obese are at risk of complications such as neonatal, perinatal, and infant death to a larger extent than babies born to women of normal weight [4, 5]. Further, in high-income countries, overweight and obesity are the most modifiable risk factors for stillbirth (population-attributional risk 8–18%), among risk factors included in a meta-analysis [6]. In addition, children of overweight or obese mothers run the risk of experiencing long-term consequences such as metabolic dysfunction and cognitive disorders [7].

Overweight and obesity during pregnancy also increases the risk of maternal complications, such as gestational diabetes, hypertensive disorders and preeclampsia [8]. According to a meta-analysis, 23.9 percent of any pregnancy complication was attributable to maternal overweight or obesity [8].

In Sweden, the number of women who were overweight and obese, when registered at a maternity clinic, is increasing. The total proportion of overweight or obese women was 25 percent in 1992 [9] and, today, 44 percent (27.6% and 16.4%, respectively) [3]. The risk of stillbirth from 37 weeks’ gestation increases according to maternal Body Mass Index (BMI 30–34: OR 1.9, BMI ≥ 35: 2.4) [10].

In order to adopt preventative measures, it is of importance to identify risk groups for obesity and negative birth outcomes. The aim of this study was to describe the sociodemographic and obstetric factors across BMI classifications; overweight, obese, and severely obese women, as compared to women of normal weight. A secondary aim was to investigate pregnancy outcomes in relation to BMI classes and gestational week.

## Methods

This is a population-based cohort study based on data from The Swedish Pregnancy Register [11]. The study includes 64,632 women, with a singleton pregnancy, giving birth from 28 weeks’ gestation. All women were registered at one of 67 maternity clinics in Stockholm, Sweden (excluded specialist maternity clinics). The included women gave birth from 1 January 2016 to 30 June 2018. The data were partly based on a cluster randomized controlled trial in which maternity clinics where randomized to intervention with the Mindfetalness-method (self-assessment method for pregnant women to observe fetal movements) or to routine care (*n* = 39,865). For this study, the time period is extended with nine months to reach a large sample size (*n* = 24,767). Further methods information is published elsewhere [12].

The 64,632 women were divided according to BMI when registered at the maternity clinic in the first trimester, defined as a person’s weight in kilograms divided by the square of the person’s height in meters (kg/m^2^) [13]. When analyzing, five groups were created; below 18.5 kg/m^2^ underweight, 18.5–24.9 kg/m^2^ normal weight, 25.0–29.9 kg/m^2^ overweight, 30.0–34.9 kg/m^2^ obesity, and 35.0 kg/m^2^ and above, severe obesity.

The study was approved by the Regional Ethics committee in Stockholm, Sweden (Dnr 2015/2105–31/1).

### Statistical analysis

Descriptive statistics (frequencies and percentages) were applied to present sociodemographic and obstetrical and baby outcomes per BMI category. Obstetrical and baby outcomes were induction of labor, cesarean section, instrumental delivery, gestational age, Apgar score, birthweight, and admittance to neonatal care unit. Obstetrical outcomes among women classified as obese or severely obese were compared with women of normal weight. We calculated risk ratio and 95 percent confidence intervals using log-binomial regression. Statistically significant differences between the compared groups were defined at the five percent level. As a final step in the analyses, we calculated the stillbirth rate according to gestational week and BMI and included only women who were still pregnant at the time of estimated birth (40 weeks’ gestation). The risk of stillbirth was calculated for women who were still pregnant at the corresponding number of weeks’ gestation, relative to their respective BMI classifications. We used statistical program R (version 3.2.4) and SPSS (version 26).

## Results

Information on BMI was obtained for 61,800 women and missing for 2832 women. The characteristics of the women are presented across five BMI-classifications (Table 1). Among women who were underweight, overweight, obese and severely obese, less women were born in Sweden and in age group 25–34 years, compared to women of normal weight. Further, women classified as being overweight/obese/severely obese had lower educational levels, were to a lesser extent employed, and were more often multiparas. The percentage of women that had a previous stillbirth increased with increased weight, with a dose–response relationship, starting from women of normal weight (0.7%), overweight women (1.3%), and obese/severely obese women (2.0%). Additionally, women overweight/obese/severely obese women were more often tobacco users at registration at maternity care. In general, overweight/obese/severely obese women were more likely to have intercurrent diseases compared to women of normal weight in a dose–response manner, according to increased weight, e.g., previous psychiatric care (Table1).

**Table 1:** Characteristics of 61,800 women, with a singleton pregnancy, giving birth from 28 weeks’ gestation, divided into Body Mass Index groups

	***BMI* < *18.5 (underweight) n* = *1729*** ***n (%)***	***BMI 18.5–24.9 (normal weight) n* = *39,000*** ***n (%)***	***BMI 25.0–29.9 (overweight) n* = *14,526*** ***n (%)***	***BMI 30.0–34.9 (obesity) n* = *4855*** ***n (%)***	***BMI* ≥ *35∫ (severe obesity) n* = *1690*** ***n (%)***
***Country of origin***					
*Sweden*	1018 (58.9)	27 441 (70.4)	8781 (60.5)	2790 (57.5)	1034 (61.2)
*Europe*	183 (10.6)	3661 (9.4)	1194 (8.2)	350 (7.2)	117 (6.9)
*Asia*	351 (20.3)	5502 (14.1)	2800 (19.3)	991 (20.4)	263 (15.6)
*Africa*	147 (8.5)	1518b (3.9)	1256 (8.6)	550 (11.3)	212 (12.5)
*South America*	15 (0.9)	572 (1.5)	380 (2.6)	141 (2.9)	50 (3.0)
*North America*	9 (0.5)	217 (0.6)	70 (0.5)	22 (0.5)	8 (0.5)
*Other*	6 (0.3)	87 (0.2)	45 (0.3)	11 (0.2)	6 (0.4)
*Missing*	0	2	0	0	0
***Age***					
≤ *24*	235 (13.6)	2621 (6.7)	1048 (7.2)	407 (8.4)	158 (9.3)
*25–34*	1143 (66.1)	25 368 (65.0)	9064 (62.4)	2949 (60.7)	1030 (60.9)
≥ *35*	351 (20.3)	11 011 (28.2)	4414 (30.4)	1499 (30.9)	502 (29.7)
***Education level***^***a***^					
*Shorter than 9 years*	33 (1.9)	337 (0.9)	335 (2.3)	165 (3.4)	79 (4.7)
*Elementary school*	108 (6.2)	1338 (3.4)	850 (5.9)	373 (7.7)	144 (8.5)
*High school*	438 (25.3)	8627 (22.1)	4541 (31.3)	1797 (37.0)	707 (41.8)
*University*	1004 (58.1)	25 678 (65.8)	7473 (51.4)	1993 (41.1)	584 (34.6)
***Occupation***^***b***^					
*Employed*	1207 (69.8)	31 157 (79.9)	10 306 (70.9)	3152 (64.9)	1082 (64.0)
*Unemployed*	51 (2.9)	906 (2.3)	497 (3.4)	205 (4.2)	78 (4.6)
*Studying*	213 (12.3)	2894 (7.4)	1366 (9.4)	501 (10.3)	154 (9.1)
*Parental leave*	94 (5.4)	2101 (5.4)	1162 (8.0)	489 (10.1)	192 (11.4)
*Sick leave*	27 (1.6)	417 (1.1)	242 (1.7)	103 (2.1)	46 (2.7)
*Other*	121 (7.0)	1308 (3.4)	830 (5.7)	347 (7.1)	120 (7.1)
***Parity***					
*Primipara*	899 (52.0)	18 302 (46.9)	5771 (39.7)	1721 (35.4)	566 (33.5)
*Multipara*	830 (48.0)	20 698 (53.1)	8755 (60.3)	3134 (64.6)	1124 (66.5)
***Previous stillbirth***	6 (0.7)	146 (0.7)	114 (1.3)	62 (2.0)	23 (2.0)
***Tobacco user at registration***	59 (3.4)	860 (2.2)	500 (3.4)	248 (5.1)	103 (6.1)
***Assisted reproduction***	81 (4.7)	2266 (5.8)	748 (5.1)	268 (5.5)	42 (2.5)
***Intercurrent diseases***					
*Diabetes mellitus*	1 (0.1)	38 (0.1)	27 (0.2)	19 (0.4)	12 (0.7)
*Coronary heart disease*	35 (2.0)	528 (1.4)	238 (1.6)	76 (1.6)	42 (2.5)
*Thrombosis*	11 (0.6)	269 (0.7)	125 (0.9)	39 (0.8)	17 (1.0)
*Psychiatric care*	174 (10.1)	4638 (11.9)	1888 (13.0)	655 (13.5)	281 (16.6)
*Endocrine disease*	82 (4.7)	2418 (6.2)	1061 (7.3)	455 (9.4)	185 (10.9)
*Chronic hypertension*	4 (0.2)	114 (0.3)	83 (0.6)	57 (1.2)	30 (1.8)
*Medication or psychological treatment for mental illness*	74 (4.3)	1945 (5.0)	899 (6.2)	343 (7.1)	101 (6.0)

^a^Missing underweight: 146 (8.4%), normal weight 3020 (7.7%), overweight 1327 (9.1%), obese 527 (10.9%), severe obese 176 (10.4%)

^b^Missing underweight: 16 (0.9%), normal weight 217 (0.6%), overweight 123 (0.8%), obese 58 (1.2%), severe obese 18 (1.1%)

Table 2 presents descriptive obstetric outcomes for women across the five BMI classifications. The higher BMI, the lower percentage of spontaneous start of labor. Correspondingly, the opposite trend is seen for labor induction, cesarean section and instrumental delivery. Prolonged pregnancy for overweight, obese or severely obese women is about the same as women of normal weight. The percentage of babies born with Apgar score of less than 10 at five minutes after birth and babies transferred to Neonatal Intensive Care Unit (NICU) increases according to the mother’s BMI. Compared to women of normal weight, babies born large for gestational age are more common among women who are overweight, obese or severely obese. The proportion of babies born large for gestational age increases by BMI.

**Table 2:** Obstetric and baby outcomes among 61,800 women with singleton pregnancy giving birth from 28 weeks’ gestation divided into BMI groups; underweight, normal weight, overweight, obesity and severe obesity

*Outcome*	*BMI* < *18.5 (underweight) n* = *1729* ***n (%)***	*BMI 18.5–24.9 (normal weight) n* = *39,000* ***n (%)***	*BMI 25.0–29.9 (overweight) n* = *14,526* ***n (%)***	*BMI 30.0–34.9 (obesity) n* = *4855* ***n (%)***	*BMI* ≥ *35 (severe obesity) n* = *1690* ***n (%)***
***Induction of labor***	267 (15.4)	6625 (17.0)	3092 (21.3)	1259 (25.9)	503 (29.8)
***Cesarean section*** *(total)*	265 (15.3)	7101 (18.2)	3285 (22.6)	1269 (26.1)	492 (29.1)
*Pre-labor*	143 (8.3)	3768 (9.7)	1634 (11.2)	609 (12.5)	210 (12.4)
*In labor*	122 (7.1)	3333 (8.5)	1651 (11.4)	660 (13.6)	282 (16.7)
***Instrumental delivery***	381 (22.0)	9525 (24.4)	3981 (27.4)	1477 (30.4)	540 (32.0)
***Preterm delivery (*< *37* + *0)***	85 (4.9)	1496 (3.8)	586 (4.0)	229 (4.7)	91 (5.4)
***Birth gestation* > *41* + *6***	76 (4.4)	2085 (5.3)	859 (5.9)	273 (5.6)	89 (5.3)
***Apgar Score* < *10 at 5 min***^***c,a***^	184 (10.7)	4686 (12.0)	1966 (13.6)	758 (15.7)	280 (16.6)
***Apgar Score* < *7 at 5 min***^***c,a***^	21 (1.2)	388 (1.0)	178 (1.2)	73 (1.5)	26 (1.5)
***Apgar Score* < *4 at 5 min***^***c,a***^	8 (0.5)	135 (0.3)	54 (0.4)	30 (0.6)	9 (0.5)
***Birthweight (mean)***^***b***^	3325.3	3507.5	3575.5	3618.1	3669.3
***Birthweight*** ≤ ***10***^***th***^ ***centile***^***d,b***^	306 (17.7)	4211 (10.8)	1374 (9.5)	399 (8.2)	110 (6.5)
***Birthweight* < *2SD***^***e,b***^	102 (5.9)	1207 (3.1)	449 (3.1)	136 (2.8)	40 (2.4)
***LGA***^***b***^	18 (1.0)	1121 (2.9)	808 (5.6)	428 (8.8)	197 (11.7)
***Admitted to NICU***	133 (7.7)	2554 (6.5)	1100 (7.6)	425 (8.8)	166 (9.8)

^a^Data are missing for underweight 10 (0.6%), normal weight 79 (0.2%), overweight 39 (0.3%), obese 16 (0.3%), severe obese 4 (0.2%)

^b^Data are missing for underweight 3 (0.2%), normal weight 38 (0.1%), overweight 12 (0.1%), obese 7 (0.1%), severe obese 5 (0.3%)

^c^Number of stillbirths (Apgar 0): underweight 2 (0.1%), normal weight 58 (0.1%), overweight 22 (0.2%), obese 18 (0.4%), severe obese 2 (0.1%)

^d^International definition of Small for Gestational Age (SGA) ≤ 10^th^ centile for the gestational age

^e^Swedish definition of Small for Gestational Age (SGA) < 2SD from the national reference mean

Large for Gestational Age (LGA)

NICU Neonatal intensive care unit

The relative risk of having a baby with Apgar score of less than four, seven and ten is higher among obese or severely obese women compared to women of normal weight (Table 3). Further, women who are obese or severely obese have higher risk of labor induction, cesarean section and instrumental delivery, compared to women of normal weight. There is twice the risk of giving birth to a stillborn baby when being obese or severely obese compared to having normal weight (Table 3).

**Table 3:** Obstetric and baby outcomes for women with singleton pregnancy giving birth from 28 weeks’ gestation; 6545 women with BMI ≥ 30 (obesity and severe obesity) versus 39,000 women with BMI 18.5-24.9 (normal weight)

***Outcome***	***BMI* ≥ *30*** *x̄* ***n (%)***	***BMI 18.5–24.9*** ***n (%)***	***Rate Ratio (95% CI)***	***P-value***
***Induction of labor***	1762 (26.9)	6625 (17.0)	1.58 (1.51 – 1.66)	< 0.001
***Cesarean section*** *(total)*	1761 (26.9)	7101 (18.2)	1.48 (1.41 – 1.55)	< 0.001
*Pre-labor*	819 (12.5)	3768 (9.7)	1.30 (1.21-1.39)	< 0.001
*In labor*	942 (14.4)	3333 (8.5)	1.68 (1.57-1.80)	< 0.001
***Instrumental delivery***	2017 (30.8)	9525 (24.4)	1.26 (1.21-1.31)	< 0.001
***Preterm delivery (*< *37* + *0)***	320 (4.9)	1496 (3.8)	1.27 (1.13-1.43)	< 0.001
***Birth gestation* > *41* + *6***	362 (5.5)	2085 (5.3)	1.03 (0.93-1.15)	0.54
***Apgar Score* < *10 at 5 min***^***e,a***^	1043 (16.0)	4701 (12.1)	1.32 (1.24-1.41)	< 0.001
***Apgar Score* < *7 at 5 min***^***e,a***^	104 (1.6)	403 (1.0)	1.54 (1.24-1.90)	< 0.001
***Apgar Score* < *4 at 5 min***^***e,a***^	44 (0.7)	150 (0.4)	1.75 (1.24-2.42)	0.002
***Birthweight (mean)***^***d,b***^	3631.9	3496.5	(-150.1**–** -120.7)	< 0.001
***Birthweight*** ≤ ***10***^***th***^ ***centile***^***f,b***^	509 (7.8)	4211 (10.8)	0.72 (0.66-0.79)	< 0.001
***Birthweight* < *2SD***^***g,b***^	176 (2.7)	1207 (3.1)	0.87 (0.74-1.01)	0.07
***LGA***^***b***^	625 (9.6)	1121 (2.9)	3.33 (3.02-3.65)	< 0.001
***Admitted to NICU***	591 (9.0)	2554 (6.5)	1.38 (1.26-1.50)	< 0.001

^a^Data are missing for obese/severe obese 15 (0.2%), normal weight 64 (0.2%)

^b^Data are missing for obese/severe obese 12 (0.2%), normal weight 38 (0.1%)

^c^Data are missing for obese/severe obese 0 (0.0%), normal weight 3 (0.0%)

^d^Welch’s *t*-test

^e^Number of stillbirths (Apgar 0) among obese/severe obese 21 (0.3%), normal weight 58 (0.1%) (RR 2.16, CI 1.31**–**3.55, *p*-value 0.006)

^f^International definition of Small for Gestational Age (SGA) ≤ 10^th^ centile for the gestational age

^g^Swedish definition of Small for Gestational Age (SGA) < 2SD from the national reference mean

Large for Gestational Age (LGA)

NICU Neonatal intensive care unit

The number of stillbirths in relation to gestational week and BMI are presented in Additional file 1, a (week 28–42) and b (including details from gestational week 40). The number of stillbirths are compared to the number of women still pregnant at 40 weeks’ gestation. Among women who were overweight, obese, or severely obese and gave birth to a stillborn baby, a higher proportion had more risk factors for stillbirth compared to women with normal weight. In the group of women who were overweight, obese, or severely obese, 46.7% (7 out of 15) were 35 years or older, compared to 26.7% in the normal weight group. Additionally, 66.7% (10 out of 15) in the group of women who were overweight, obese, or severely obese were born outside Sweden, compared to 40.0% (6 out of 15) in the normal weight group. There is a double risk for a woman who is overweight to give birth to a stillborn baby from gestational week 40, compared to women of normal weight (*n* = 8/7271 vs. *n* = 15/19,783, RR 2.06, CI 1.01–4.21. *p*-value 0.05). Additionally, there is an almost four times higher risk for women who are obese to give birth to a stillborn baby from gestational week 40, compared to women of normal weight (*n* = 15/9599 vs. *n* = 15/19,783, RR 3.97, CI 1.6–9.7, *p*-value 0.006). The number needed to treat (NNT) (labor induction before 40 weeks’ gestation) births among women classified as overweight/obese/severely obese is 1.45/1000 (15 babies if inducing 10,368 women). Among women of normal weight, the corresponding figure is 0.76/1000 (15 babies if inducing 19,783 women).

## Discussion

From 40 weeks’ gestation, women classified as overweight have a double risk of stillbirth compared to women of normal weight, and the risk increased to almost four times higher for obese women. Women who were obese or severely obese had higher risk of almost all adverse pregnancy outcomes such as low Apgar score, stillbirth, transfer to neonatal care and instrumental delivery, compared to women of normal weight.

A recent study with data from the United States confirms the dose–response risk for stillbirth according to BMI and gestational week [14]. Obesity has a causal relationship to various adverse pregnancy outcomes, such as stillbirth, and is one of the most important factors to focus on for prevention [6]. Additionally, according to the risk of perinatal mortality, there is a curve-linear relationship, with higher risks in obese women from 39 week’s gestation, compared to women of normal weight (the longer the pregnancy progresses from 39 gestational weeks, the greater the risk for the obese). The underlying mechanisms for higher risk of stillbirth among overweight/obese women are still unclear. It is suggested that one cause for unexplained stillborn babies to women with higher BMI is that they are discretely small-for-gestational-age [15] and studies show higher risk of small-for-gestational-age by increasing BMI [16]. In a Chinese population, obesity has been shown to be a risk factor for small-for-gestational-age babies (RR 2.66, CI 2.01–3.52) [17]. The risk for giving birth to a small-for-gestational-age infant can also be linked to transgenerational transmission [18]. An association is seen between placenta-mediated diseases, such as small-for-gestational-age and preeclampsia, and epigenetic factors that can be transferred to subsequent generations. The risk of having a small-for-gestational-age baby increases by almost three times if the mother has a small-for-gestational-age background herself [19]. Insulin resistance, endothelial dysfunction, oxidative stress, lipotoxicity, inflammation, and infection are some possible mechanisms behind the higher risk for obstetrical complications for women who are overweight/obese. Obese women also have an increased risk of hypertension, preeclampsia, and impaired placental function, which can also be contributing factors for the higher risk of having a small-for-gestational-age baby or stillbirth [20]. A high-fat diet may lead to dysfunction of placenta and higher risk of stillbirth, as seen in studies on primates and sheep [21, 22]. A reduction in uterine volume blood flow and increased placental inflammatory cytokines were seen among primates with intake of a high-fat diet.

In obstetric care the healthcare professionals weigh the pros and cons when inducing labor before the due date, with the aim of preserving the pregnancy, if possible. The advantages of inducing labor before the due date might save some babies’ lives, but the negative aspects are the medicalization of normal pregnancies and a risk of negative consequences for mother and baby in the short- and long-term perspective [23]. Almost 50% of the women who were overweight/obese in our study were still pregnant when reaching 40 weeks’ gestation. Further, among women with stillbirth from 40 weeks’ gestation, a higher percentage were overweight or obese and additionally had two more risk factors for stillbirth, such as advanced maternal age and country of birth outside Sweden. This underlines the importance of considering inducing women having a risk pregnancy, if they pass their due date. In our data, according to number needed to treat among women with BMI from 25, compared to women of normal weight, a lower proportion of women must be induced from 40 weeks’ gestation to prevent one stillbirth (15 stillbirths inducing 10,368 women versus 19,783 women). If inducing all women who are overweight/obese from 40 weeks’ gestation, some babies in this study (approximately six), conducted in the capital of Sweden, could have been saved. Extrapolating these to numbers nationally, about 20 babies can be saved per year (Stockholm has 26% of all births in Sweden). However, we do not have information about the causes for death for the stillbirths in our study. It is possible that some of the babies who were stillborn from 40 weeks’ gestation could not be saved by an earlier induction of the delivery. Additionally, many diseases, for example diabetes, covariates with several other factors, such as ethnicity, which needs to be taken into account, as well as other confounding factors.

Our study confirms earlier research that obese and severe obese women have a higher risk of having severe obstetric and baby complications [24, 25]. Preventive methods in reducing overweight/obesity among young women is important [26, 27]. Beside public health policies, more resources must be allocated to youth centers [28], childcare centers and schools.

In the Stillbirth Lancet series, researchers identify overweight and obesity as important modifiable risk factors for stillbirth and stress that action is needed for prevention [6, 29]. Preferably, preventive methods should start at an early age to reverse the development that is taking place. If young women, for example adolescents in schools and at youth centers, are educated about health and pregnancy at an early stage, healthy diet and physical activity should be some of the areas to discuss. By informing women about the impact of these factors on pregnancy and overall health, and by giving support for healthy choices their risk of being overweight/obese when pregnant might be reduced. Additionally, if a woman shows interest in receiving further help in adhering to a healthier lifestyle, this woman can be identified and referred to other suitable professionals [30].

When investigating the 21 healthcare regions in Sweden, differences are seen in the guidelines for the management of women who are overweight and obese, when registered at a maternity clinic [11]. The guidelines differ in when or whether a growth ultrasound should be conducted: 13 regions follow the same guidelines as if normal weight and in eight regions ultrasound is indicated if BMI > 35. Further, in seven regions an oral glucose tolerance test is indicated if BMI > 30 and in 13 regions if BMI > 35. The guidelines also differ in terms of whether a medical doctor or a dietician should be consulted and only 52 percent of the regional guidelines indicate that extra visits to the midwife is needed if the women are overweight/obese. It might be advantageous to have national, individualized care for women with BMI of 25 or above, as risk for negative obstetrical outcomes is linear according to BMI, and even modest increases in maternal BMI are associated with increased risk of stillbirth, perinatal and neonatal death [4]. Further, the national guidelines should adapt to the clinical recommendations by FIGO (The International Federation of Gynaecology and Obstetrics) and health care policies should target women in a prepregnancy stage in primary care, as suggested in the recommendations by FIGO [31]. Recently, NICE guidelines in the United Kingdom recommend labor induction from 41 weeks’ gestation and further recommend more research focusing on women with BMI of over 30 and women aged over 35 years which are groups of women who may be more likely to experience adverse outcomes if their pregnancy continues [32]. This actual study clearly indicates that overweight and obese women need to be monitored extra closely during pregnancy and individually assessed for induction of labor versus close monitoring at term.

### Strengths and limitations

A strength of this study is that the data are drawn from the population-based register, including a large number of data and almost all women giving birth in Stockholm. Further, there is a low percentage of missing values.

The study also has some limitations. In Sweden, there are regional differences in the prevalence of women with overweight and obesity. According to Chaparro et al. [33], Stockholm, which has been studied in this cohort, has the lowest prevalence. This would mean that the percentage of women who are overweight/obese is even higher in other parts of Sweden. The generalizability of the results is high for large cities in Sweden such as Stockholm, but less to other parts. However, this would mean that negative outcomes for women in rural areas could be even more pronounced.

We do not know the causes for stillbirth in this study. Additionally, we have no information on why the women who were obese were still pregnant around their due date. Some of the women could have been offered induction of labor but declined. Even if the risk of stillbirth increases by being overweight and prolonged pregnancy, the outcome is rare, and the woman’s choice is important in person-centered care.

The aim of the study was to identify sociodemographic factors and investigate outcomes related to BMI; we have not adjusted for potential confounders. When comparing obese women with women of normal weight, it is possible that other factors affect the outcomes among obese women. The comparing groups differ in sociodemographic factors, such as country of origin, educational level, previous stillbirth, tobacco use and diseases, factors that may have affected the outcomes.

## Conclusion

Women who were obese or severely obese had a higher risk of almost all adverse pregnancy outcomes. These findings highlight the need for national obesity preventive strategies at a young age. Further, in maternity care there is a need for national guidelines and individualized care for women with overweight/obesity. Overweight and obese women need to be monitored extra closely during pregnancy and individually assessed for induction of labor versus close monitoring at term, in consultation with the woman.

## Supplementary Information

**Additional file 1. ** Number of stillbirths in relation to gestational week and Body Mass Index (BMI) among 61,800 women.

## Data Availability

The Ethics committee prohibit data to be publicly available due to confidential information. However, the data will be shared after an approval from the Regional Ethics committee in Stockholm, Sweden (https://etikprovningsmyndigheten.se/) by contacting registrator@etikprovning.se.

## Abbreviations

BMI: Body Mass Index
RR: Relative Risk
NICU: Neonatal intensive care unit
NNT: Number Needed to Treat
