# Supplementary material for: Prolonged pregnancy and stillbirth among women with overweight or obesity – a population-based study in Sweden including 64,632 women
Source: BMC Pregnancy Childbirth. 2023 Jan 12;23:21. doi: 10.1186/s12884-022-05340-4 (PMC9835339; doi:10.1186/s12884-022-05340-4)
Supplement: Supplementary file 1 — Additional file 1. Number of stillbirths in relation to gestational week and Body Mass Index (BMI) among 61,800 women. [file 12884_2022_5340_MOESM1_ESM.docx]

**Additional file 1a.** Number of stillbirths in relation to gestational week and Body Mass Index (BMI) among 61,800 women.

| **GW** | **BMI <18.5**  **(underweight)**  **n=1729** | **BMI 18.5–24.9**  **(normal weight)**  **n=39,000** | **BMI 25.0–29.9**  **(overweight)**  **n=14,526** | **BMI 30.0–34.9**  **(obesity)**  **n=4855** | **BMI ≥35∫**  **(severe obesity)**  **n=1690** |
| --- | --- | --- | --- | --- | --- |
| 28-31+6 | 1 | 11 | 3 | 2 | 2 |
| 32-36+6 | 1 | 23 | 5 | 1 | 0 |
| 37 | 0 | 9 | 2 | 2 | 0 |
| 38 | 2 | 3 | 3 | 5 | 0 |
| 39 | 0 | 6 | 3 | 2 | 0 |
| 40 | 0 | 8 | 5 | 5 | 0 |
| 41 | 0 | 6 | 2 | 2 | 0 |
| 42+ | 0 | 1 | 1 | 0 | 0 |
| Percent | 0.0023 | 0.0017 | 0.0017 | 0.0039 | 0.0012 |

**Additional file 1b.** Stillbirth among women in gestational week (gw) 40**–**42* in relation to Body Mass Index (BMI).

| **GW** | **BMI <18.5**  **(underweight)**  **n=776**  **n/n (%)** | **BMI 18.5–24.9**  **(normal weight)**  **n=19,783**  **n/n (%)** | **BMI 25.0–29.9**  **(overweight)**  **n=7271**  **n/n (%)** | **BMI 30.0–34.9**  **(obesity)**  **n=2328**  **n/n (%)** | **BMI ≥35∫**  **(severe obesity)**  **n=769**  **n/n (%)** |
| --- | --- | --- | --- | --- | --- |
| 40 | 0/776 (0) | 8/19 783 (0.0004) | 5/7271 (0.0007) | 5/2328 (0.002) | 0/769 (0) |
| 41 | 0/317 (0) | 6/8944 (0.0007) | 2/3432 (0.0006) | 2/1091 (0.002) | 0/374 (0) |
| 42 | 0/76 (0) | 1/2085 (0.0005) | 1/859 (0.001) | 0/273 (0) | 0/89 (0) |
| Total | 0/776 (0) | 15/19783 (0.0008)∞ | 8/7271 (0.0011)∫ | 7/2328 (0.0030)≠ | 0/769 (0) |

*Gestational week: number of women pregnant in gestational week 40 and number of women of those still pregnant in gestational week 41 and 42 *n*/*n*.

∞ Country of birth outside Sweden *n*=6 (40%), Age >35 *n*=4 (26.7%)

∫ Country of birth outside Sweden *n*=4 (50%), Age >35 *n*=3 (37.5%)

≠ Country of birth outside Sweden *n*=6 (85.7%), Age >35 *n*=4 (57.1%)
